# Spatial differentiation of gully clusters based on the regional scale: an example from northeastern China

**DOI:** 10.7717/peerj.9907

**Published:** 2020-10-19

**Authors:** Ying Zhao, Bin Zhang, Wei Qin, Jun Luo, Hui Liu, Qingchun Deng, Wei Lv, Yuli He, Houling Wang

**Affiliations:** 1 Sichuan Provincial Engineering Laboratory of Monitoring and Control for Soil Erosion on Dry Valleys, China West Normal University, Nanchong, China; 2 School of Land and Resources, China West Normal University, Nanchong, China; 3 State Key Laboratory of Simulation and Regulation of Water Cycle in River Basin, China Institute of Water Resources and Hydropower Research, Beijing, China; 4 Research Center on Soil and Water Conservation of the Ministry of Water Resources, Beijing, China

**Keywords:** Gully, Spatial cluster, Kernel density, Regional differentiation, Azonality

## Abstract

Gully erosion represents a serious environmental threat around the world, but their spatial distribution law are unclear at the regional scale. To quantitatively characterize the spatial distribution model of gullies and determine the regularity of regional differentiation, this paper used spatial analyst and statistics method to study the spatial distribution of gullies in 34 sample areas of northeastern China based on interpretations of high-resolution remote sensing images. The results showed that the kernel density could quantitatively describe the continuous spatial clustering of gullies. Gullies in northeastern China had the characteristics of a spatially unbalanced distribution at the scale of the sample area. The average kernel density of the 34 sample areas (Moran’s *I* was 0.43, P¡0.01*P* < 0.01) also indicated clustering distribution at the regional scale. The horizontal clustering characteristics of gullies exhibited an azonal distribution of being low values in the middle plain and high values on the three mountainous areas. The average kernel density in the southeastern part of the study area was highest (maximum value of 2.38). In the vertical direction, gullies were relatively undeveloped in low- and high-altitude areas, while middle-altitude areas were beneficial to the development of gullies. The effect of height differences on gully development was more significant than altitude. As the height difference increased, gullies tended to be more clustered, which can be expressed by a power function. The results of this study will not only help to understand the regional differentiation characteristics of gullies but will also provide a scientific reference for the study of spatial distribution of gullies in future.

## Introduction

Soil erosion is one of the most severe environmental issues in the world. Globally, an area of 16.42 × 10^6^ km^2^ is affected by soil erosion; thus, it is considered to be the main cause of global land degradation (Valentin, Poesen & Li, 2005). Gully erosion is one of the most common types of water erosion, and can lead to environmental disasters (Gao et al., 2013; Wu et al., 2008). On a hillslope, runoff concentrates and wash away the soil and parent material, then cuts into the surface to form gullies. Gullies can be classified as either ephemeral or permanent (Poesen et al., 2003), and their development is often controlled by multiple factors (Deng et al., 2015; Ran et al., 2018).

Studies of gully distribution began with an investigation of five gully types in a 400 km^2^ area by Thomas in the mid-1950s. However, progress has been slow in the following 50 years. In the past 20 years, the spatial pattern of gullies has become a research focus for gully geomorphology. With the development of GIS and RS technology, gully inventory can be extracted from high-accuracy image of Google Earth, and GIS can be used to process the images, to obtain information for characterizing gully spatial pattern and gully mapping (Bogale et al., 2020; Frankl et al., 2013; Karydas & Panagos, 2020). In recent years, based on 3S technologies, pioneering research has been conducted on the spatial pattern and distribution of gullies and their topographic differentiation in the Loess Plateau (Tang et al., 2015; Yi et al., 2016) and black soil region of northeastern China (Huang & Fan, 2017; Wang et al., 2008). The distribution of gullies at different spatial scales in China is inhomogeneous, and, at the national scale, they are concentrated mainly in the Loess Plateau, northeastern China, and the southwestern dry-hot valleys (Deng et al., 2015; Ran et al., 2018).

The spatial distribution of gullies is mostly characterized by gully density (Golosov et al., 2018; Gurbanov & Ganieva, 2017; Guyassa et al., 2018; Li et al., 2007; Litvin et al., 2003), with an administrative area or watershed usually taken as the evaluation unit. Density mapping can express the spatial distribution of gullies (Arabameri et al., 2018; Golosov et al., 2018). Gully density has characteristics of horizontal differentiation, vertical (elevation) differentiation, and slope and slope direction differentiation (Li et al., 2017; Litvin et al., 2003; Wang et al., 2008; Xu et al., 2014). Gully density in the Loess Plateau is greatest in Suide, Liulin, Wubao, and Linxian areas (the gully density is greater than 10.0 km/km^2^) (Tian et al., 2013), while the Yuanmou Basin has the greatest concentration of gullies in the Jinsha River (dry-hot) Valley (Chai, Fan & Liu, 2001; Xu et al., 2018). This spatial differentiation is affected by multiple factors, such as rainfall, vegetation coverage, topography, soils, land use, and human activities (Deng et al., 2020; Gurbanov & Ganieva, 2017; Karydas & Panagos, 2020; Luffman & Nandi, 2020). The dominant factors affecting the distribution of gullies may differ among regions. For example, vegetation coverage and topography are the main factors controlling gully density in the Loess Plateau (Zhao et al., 2016). Gully density in the black soil region of northeastern China changes regularly with changes in precipitation (Fan, Zhen & Wang, 2015), whereas the correlation between gully density and annual rainfall in the Hengduan Mountains is weak (Dong, Nie & Xiong, 2018). A change of land use may be an important factor in triggering gully erosion. This has been observed in the transformation of forestland to cultivated land and grassland in northeastern China, where the gully density has increased rapidly (Yan, Zhang & Yue, 2006). Topographic factors have complex effects on the distribution and density of gullies, with gully density reported to increase as the slope increases (Dong, Nie & Xiong, 2018; Wang, Fan & Fan, 2017), or to initially increase and then decrease (Chen et al., 2016; Wang & Fan, 2019; Xu et al., 2014; Yan, Zhang & Yue, 2007). When considering different slope directions, the gully density on sunny slopes is higher than on shady slopes, and is higher on windward slopes than on leeward slopes (Li, Zhang & Li, 2012; Yan, Zhang & Yue, 2007; Zhang et al., 2015). The probability of gullies forming on a concave slope is almost twice that for a convex slope, with a lower probability of gullies forming on a straight slope (Chen et al., 2016; Li, Zhang & Li, 2012). In contrast, a convex slope is most conducive to the formation of gullies under shady conditions (Li, Zhang & Li, 2012). Gully density is also closely related to the catchment area or watershed, and decreases as the ratio of basin length increases (Xu et al., 2018). Lithology is one of the decisive parameters affecting the formation of gullies (Guyassa et al., 2017; Poesen et al., 2003). Each material has a different permeability and shear resistance, with sandstone often being conducive to the development of a high density of gullies (Rahmati et al., 2017).

The spatial distribution of gullies at the watershed scale has been widely investigated based on gully density. However, gully density depends on specific regional units (such as administrative areas), which cannot reflect internal differences and characterize the continuous distribution of gullies at the regional scale. The main objective of this study was to explore the spatial pattern of gully distribution at the regional scale. The results will help to understand the spatial characteristics and mechanisms that influence the distribution pattern of gully at the regional scale.

### The study area

**Figure 1:**
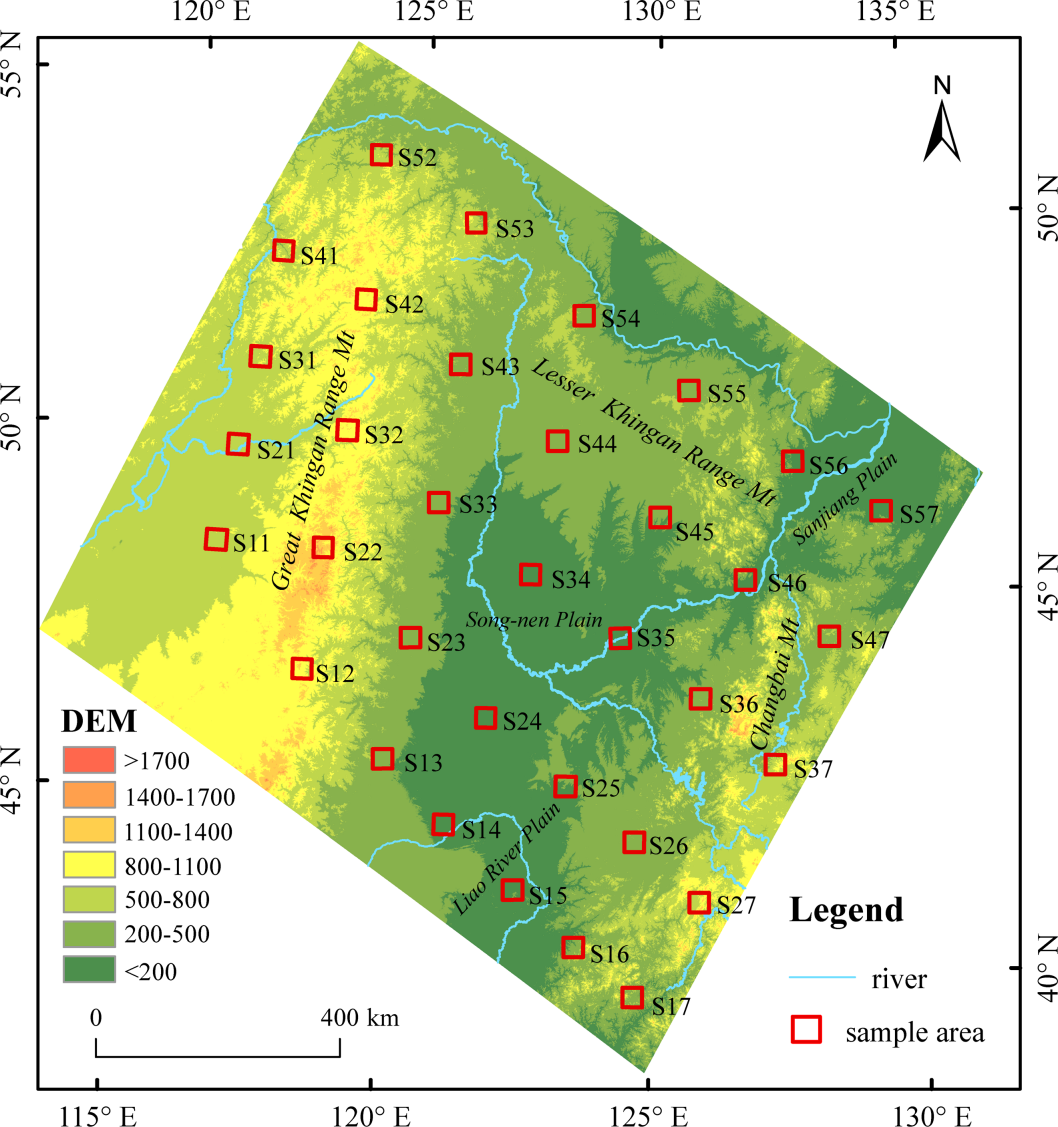
Location of the samples area. Map credit: Bin Zhang.

The study area is located in northeastern China (38°30′∼53°33′N, 118°53′∼135°05′E), including the administrative areas of Heilongjiang Province, Jilin Province, Liaoning Province, and the eastern part of the Inner Mongolia Autonomous Region (Fig. 1). The area has a monsoon climate that is typical of medium latitudes with four distinct seasons. Rainfall is concentrated in the summer, the annual precipitation is 300–1,000 mm, and the annual average temperature is −5∼11 °C. The mountains of the Greater Khingan Range, Lesser Khingan Range, and Changbai surround the study area to the west, north, and east, respectively, and the Plains of the Liao River, Song-nen Rivers, and Sanjiang lie in the middle. The vegetation coverage changes from north to south with the change in caloric conditions, varying from coniferous forest to temperate coniferous and broad-leaved mixed forest, and finally warm temperate deciduous broad-leaved mixed forest. From east to west the vegetation coverage changes with the change in humidity, varying from forest to meadow steppe (forest steppe), and, finally, to a typical grassland landscape (Li, Zhao & Zhou, 2019; Xu, 1986). Vegetation coverage is high in northeastern China, and higher coverage in the east is distinct. Large areas of dark brown soil are distributed in the mountains, including forest soil and bleached gray soil in the west of the Greater Khingan Range. Black soil, chernozem, planosol, and meadow soil are found in the Song-nen River Plain, and chernozem, chestnut soil, meadow soil, bog soil, and aeolian sandy soil are found in the Liao River Plain. The Sanjiang Plain contains black soil, planosol, meadow soil, and bog soil. The soil in the Liaodong Hilly Area is composed mainly of brown soil. Due to the special geographical environment and the influence of human activities, gullies in northeastern China are relatively well developed. The large gullies in these areas could pose a threat to food security in China, and so to prevent gully expansion is necessary.

## Data and Methods

### Sample selection

Due to the large size of the territory, it is quite difficult to accurately interpret the gullies throughout the study area. The selection of appropriate sample areas can lead to an effective alternative method. The arrangement of the belt transects was either parallel or perpendicular to the ranges of mountains, which was coincident with the prevailing wind direction of the area. Five belt transects were laid in the northwest–southeast direction (near east–west direction) and seven in the northeast–southwest direction (near north–south direction). The near east–west belt transects were numbered WE1, WE2, WE3, WE4, and WE5 from south to north, and the near south–north belt transects were numbered NS1, NS2, NS3, NS4, NS5, NS6, and NS7 from west to east. The intersection of the belt transects in the two directions was the center of the sample area. The sample area had a square side length of 30 km with a near north–south or east–west orientation, and a total of 35 sample areas were available. The sample numbering was based on the S*ij* format, where *i* is the near east–west transect number (1–5), and *j* is the near south–north transect number (1–7). Because sample area S51 locates outside of China, it was excluded from the study, leaving 34 sample areas to be considered.

### Interpretation of gullies

**Figure 2:**
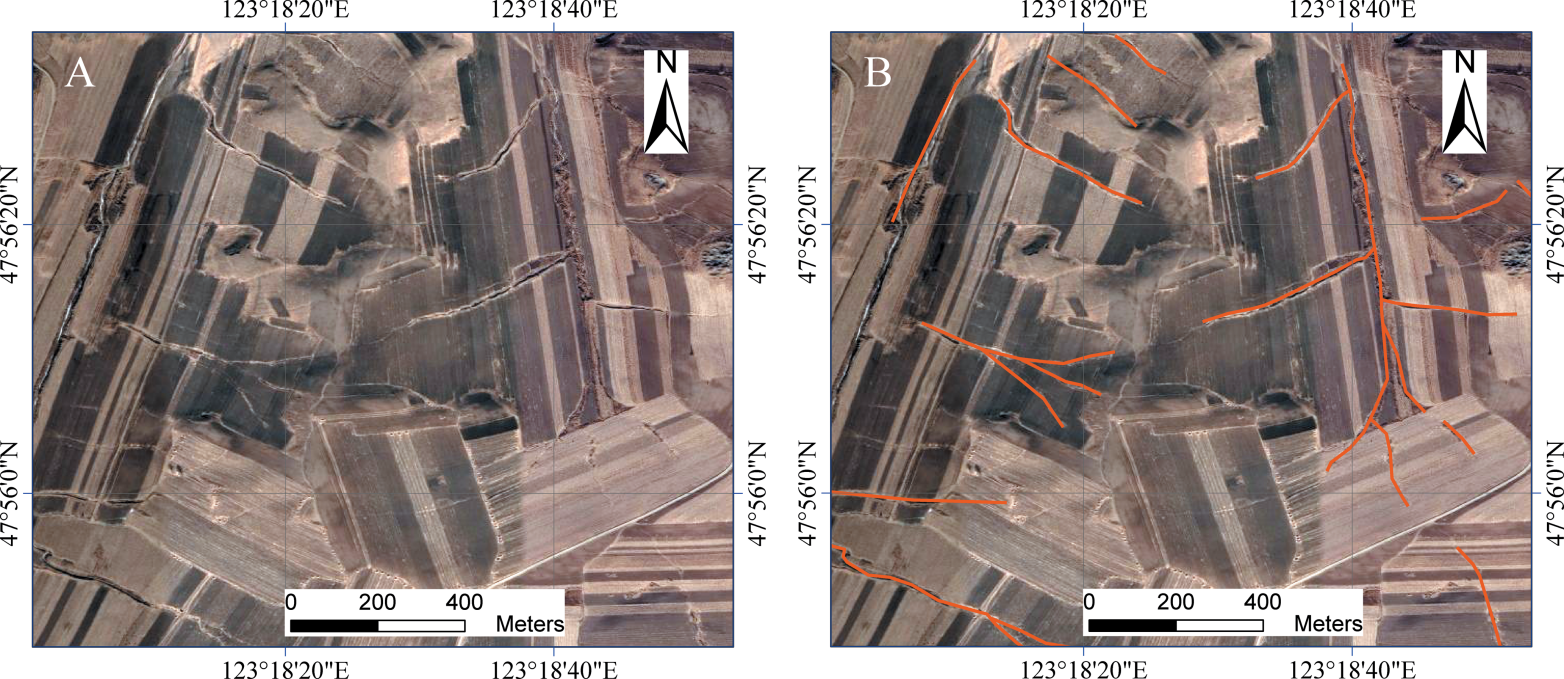
The gullies interpretated (A: original image; B: image with gullies). Image from Google Earth (Imagery: ©2019 Google, DigitalGlobe).

Based on the high-resolution images by Google Earth attained in recent five years with spatial resolution of less than 2.0 m, the visual interpretations of gullies were conducted in ArcGIS software (version 10.4). An interpretation mark was created based on image tone, veins, contours, morphology, direction, topography, and vegetation status (Golosov et al., 2018; Okwu-Delunzu et al., 2013; Wang et al., 2016). The gully thalwegs were digitized as polyline feature (linear elements) in ArcGIS (Fig. 2); then the field of length was added in the attribute table of polyline feature and calculated by the tool of Calculate Geometry; finally the gullies with length of less than 200 m were removed and 35,463 gullies were identified. We used Shuttle Radar Topography Mission (SRTM) DEM data with a resolution of 90 m, which was originally produced by NASA. The average, minimum, and maximum values of the DEM in the sample areas were calculated. The elevation of the sample area was represented by the average value, and the difference between the maximum and minimum values represented the height difference in the sample area. In this study, GCS_WGS_1984 was used for the geographic coordinate system of remote sensing images and DEM.

### Kernel density evaluation (KDE)

KDE is the most widely used estimation method in spatial pattern analysis. It is also an effective technique for measuring local density variations and exploring the continuous distribution of events. The kernel density (KD) has an advantage over the gully density with characteristics of continuous and unequal distribution of gullies in a region, but the gully density can only be a value in this region. The KDE of gullies was conducted using the Rosenblatt-Parzen formula (Parzen, 1962; Rosenblatt, 1956; Ruppert & Cline, 1994):

(1) \begin{eqnarray*}{f}_{n}(x)= \frac{1}{nh} \sum _{i=1}^{n}k( \frac{x-{X}_{i}}{h} )\end{eqnarray*}

where *n* is the number of gullies; *h*> 0, which is the bandwidth; *k*(⋅) is the kernel function; and (*x*−*X*_*i*_) represents the distance from the estimation point to event *X*_*i*_. The larger the KD, the denser the gully in the area, and the higher the probability of the occurrence of an event. According to the ArcGIS, the bandwidth (*h*) was calculated as follow:

(2) \begin{eqnarray*}h=0.9\times \min \nolimits \left( \mathrm{SD},\sqrt{ \frac{1}{ln2} }\times Dm \right) \times {n}^{-0.2}\end{eqnarray*}

where *Dm* is the median of these distance, and SD is the standard distance.

The spatial distribution of the KD values of gullies in the 34 sample areas was calculated in ArcGIS with the polyline feature, and the average kernel density (AKD) of each sample area was then calculated. The AKD was used to characterize the overall spatial distribution of gullies in the sample area, and the maximum KD to the most intensive distribution in local part of this region.

### Spatial distribution pattern recognition

Moran’s *I* index is a description of the spatial characteristics of a certain phenomenon or attribute value in the whole region to check whether there is clustering, dispersion or randomness in the space (Kumari, Sarma & Sharma, 2019; Zhan et al., 2016), which was used to analyze the degree of clustering and dispersion of AKD in the 34 sample areas, which determines whether the KD of the 34 sample areas was spatially clustered. The formula is:

(3) \begin{eqnarray*}{\mathrm{Moran}}^{{^{\prime}}}\mathrm{s}~I= \frac{n\sum _{i=1}^{n}\sum _{j1}^{n}{W}_{ij}({x}_{i}-\bar {x})({x}_{j}-\bar {x})}{\sum _{i=1}^{n}\sum _{j=1}^{n}{W}_{ij}\sum _{i=1}^{n}({x}_{i}-\bar {x})^{2}} ,\mathrm{i}\not = \mathrm{j},\end{eqnarray*}

where *n* refers to the number of sample areas; *x*_*i*_ and *x*_*j*_ refer to the KD values of sample areas *i* and *j*, respectively; $\overline{x}$ refers to the average of x; and *W*_*ij*_ is the spatial weight matrix element. Moran’s *I* has a value range of [−1, 1]. When *I* is positive, the spatial KD has a clustering characteristic; when *I* is negative, the spatial KD has a discrete characteristic; and when *I* is 0, the space is not correlated and the KD is characterized by a random distribution.

## Results

### Gully distribution characteristics

#### Statistical characteristics

**Figure 3:**
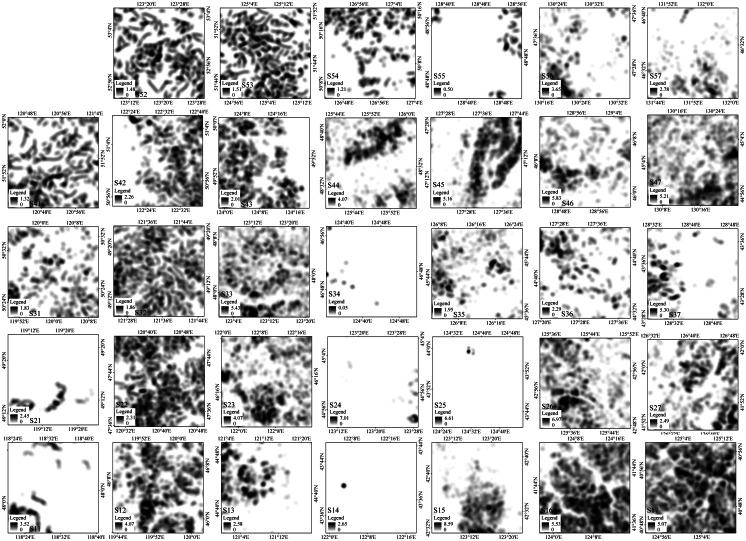
Kernel density (KD) distribution of the sample areas (black means high KD; white means low).

The spatial continuous distribution of gullies was characterized by the method of KDE (Fig. 3). The results of KD evaluation showed that gully distribution was not uniform at the sample scale. Of the 34 sampled areas, only five had a maximum KD of less than 0.1, indicating that the gullies in these areas were extremely underdeveloped. The range of maximum KD values was 0.05–8.59. There were four samples areas (S15, S24, S25, and S26) with a maximum KD of greater than 6.0, while ten sample areas had values between 4.0 and 6.0, fourteen between 2.0 and 4.0, and nine sample areas had KD values of less than 2.0. In particular, there were two sample areas with a maximum KD of less than 1.0. From the AKD, there were two sample areas with KD values of greater than 2.0, seven areas with values of greater than 1.0 (Table 1), and six areas with values of less than 0.1. Therefore, there were large differences in the development of gullies in each sample area, and their distribution had unbalanced characteristics.

**Table 1:** Statistical characteristics of the gullies in the sample areas.

ID	Center longitude	Center latitude	KD			Elevation (m)			Height difference (m)
			Min	Max	Average	Min	Max	Mean	
S11	118.570600	48.017998	0.000	3.524	0.149	618	807	716	189
S12	119.910779	46.077695	0.000	4.067	1.093	721	1262	932	541
S13	121.203409	44.684990	0.000	2.583	0.355	197	560	269	363
S14	122.152479	43.657390	0.000	2.649	0.001	167	224	182	57
S15	123.233768	42.607187	0.000	8.592	0.697	62	431	106	369
S16	124.155689	41.681920	0.000	5.530	2.381	93	856	318	763
S17	125.050768	40.855175	0.037	5.068	2.350	146	1105	410	959
S21	119.293426	49.288552	0.000	2.451	0.076	579	706	619	127
S22	120.706217	47.689352	0.000	2.312	0.988	888	1674	1223	786
S23	122.146670	46.261033	0.000	4.072	1.002	275	508	354	233
S24	123.332692	44.989880	0.000	7.011	0.062	111	162	141	51
S25	124.605923	43.859454	0.000	6.613	0.003	140	290	189	150
S26	125.681803	42.922948	0.000	6.967	1.289	311	655	377	344
S27	126.641308	41.929011	0.000	2.493	0.615	375	1484	777	1109
S31	120.032134	50.456997	0.000	1.826	0.411	554	1032	703	478
S32	121.593975	49.243599	0.000	1.856	0.866	769	1272	946	503
S33	123.194292	48.030777	0.015	5.429	1.226	200	474	292	274
S34	124.751729	46.809291	0.000	0.050	0.005	130	162	145	32
S35	126.244578	45.696281	0.000	1.950	0.310	102	185	129	83
S36	127.528025	44.642866	0.000	2.290	0.440	194	609	265	415
S37	128.644279	43.531859	0.000	5.296	0.476	349	1224	666	875
S41	120.875951	51.878845	0.003	1.322	0.550	415	1150	691	735
S42	122.458809	51.004130	0.000	2.255	0.634	729	1254	945	525
S43	124.181605	49.865099	0.000	2.007	0.709	295	632	397	337
S44	125.843013	48.545180	0.000	4.065	0.731	221	483	277	262
S45	127.557477	47.203251	0.000	5.160	0.966	176	284	216	108
S46	128.934200	46.090084	0.000	5.832	0.706	92	843	192	751
S47	130.269880	45.055773	0.094	5.211	1.489	269	727	425	458
S52	123.348403	52.984185	0.004	1.476	0.548	336	904	592	568
S53	125.102601	51.772658	0.000	1.506	0.605	365	1031	620	666
S54	126.948335	50.175165	0.000	1.206	0.334	203	607	390	404
S55	128.732949	48.825058	0.000	0.500	0.068	285	523	403	238
S56	130.446257	47.519862	0.000	3.653	0.508	80	400	150	320
S57	131.909682	46.540431	0.000	2.783	0.304	59	550	115	491

#### Distribution model

**Figure 4:**
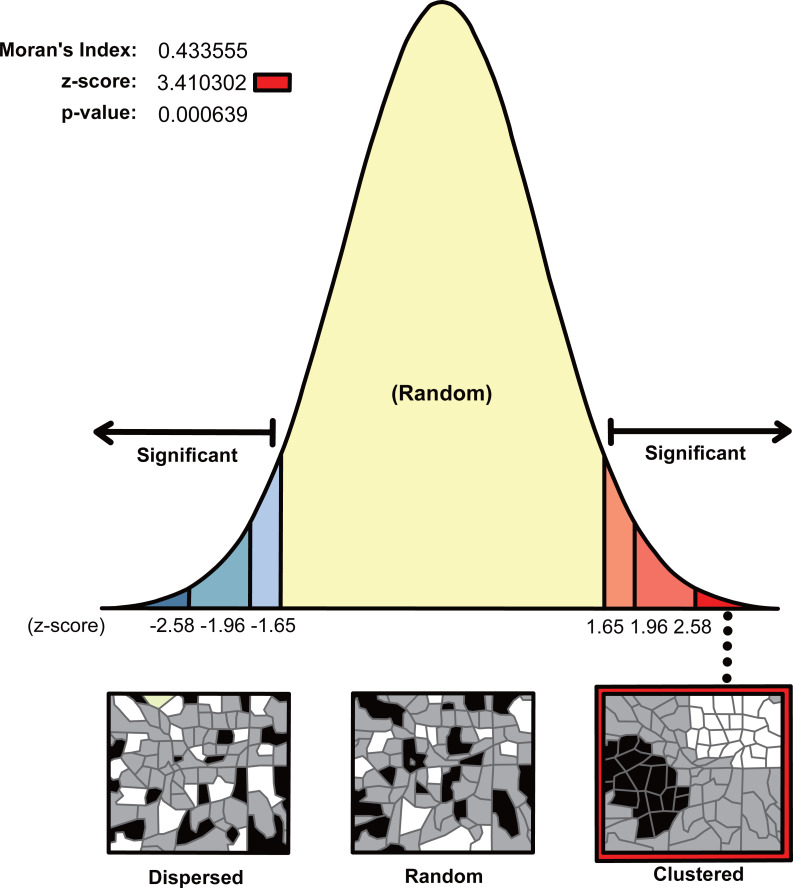
Distribution model of AKD in the 34 sample areas.

The spatial autocorrelation statistical analysis of AKD of the 34 sample areas produced a Moran’s *I* value of 0.43 (Fig. 4), indicating that the KD distribution of the gullies presented inhomogeneity at the regional scale as well as a clustering pattern. The *Z* score was high, and the *P* value was less than 0.01, which means that the probability of the data having a random pattern was low and the confidence level was higher than 99%. The spatial distribution of AKD revealed a concentrated distribution of high- and low-value areas.

### Horizontal differentiation

**Figure 5:**
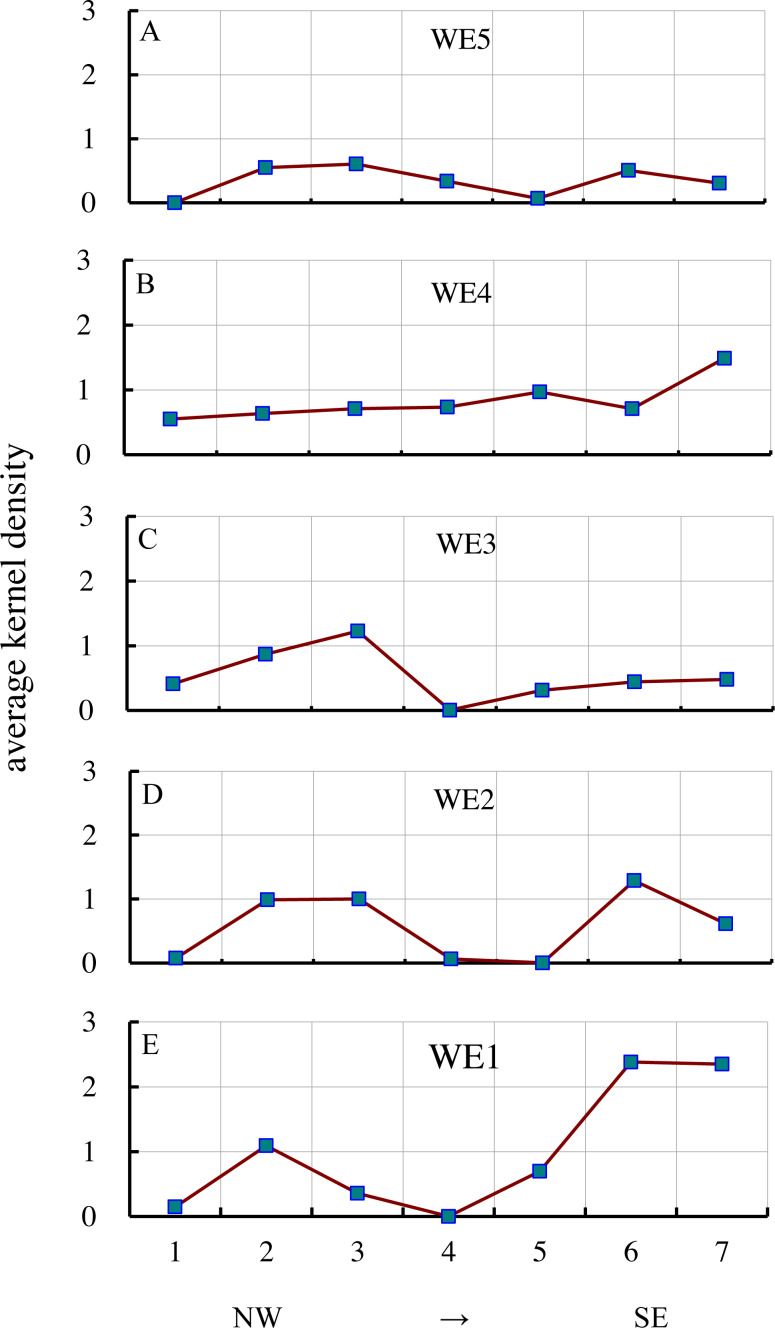
Variations in AKD from NW to SE. A, B, C, D & E represent WE5, WE4, WE3, WE2 and WE1, respectively, indicating the AKD change from the NW to SE of a near east–west belt transect.

The AKD of gullies varied from northwest to southeast (Fig. 5). The three southernmost series (WE1, WE2, WE3) had low values in the middle, while the two sides had high values, i.e., AKD in the northwest and southeast was relatively high, while in the central area it was relatively low. In the southern sample area series (WE1), from northwest to southeast, the first sample area (S11) had a low AKD of 0.15, which increased to 1.09 (S12) and then decreased to 0.36 (S13); there were almost no gullies in the sample area S14. In the southeastern direction, AKD rose from 0.70 (S15) to 2.38 (S16) to 2.35 (S17). The AKD of gullies to the southeast was significantly higher (twice) than that to the northwest. The series in the second sample area from south to north (WE2) displayed almost the same pattern of variation as the southernmost series, but the difference between the AKD of gullies in the southeast and northwest was relatively small. In the third sample area series (WE3), the AKD values of the three sample areas (S35, S36, S37) in the southeast were lowest, with a slight increase further southeast. The maximum AKD (1.23) in the northwest was significantly higher than the maximum in the southeast (0.48). The fourth series of sample areas (WE4) differed greatly from the three sample area series in the south. With the exception of S46, there was an obvious increase from the northwest to the southeast, with the lowest value of 0.55 in the northwest (S41), increasing to 1.49 (S47). In the fifth sample area series (WE5), the AKD values of the six sample areas were lower, with the maximum value being only 0.61, and a general pattern of low values in the central area and high values in the northwest and southeast.

**Figure 6:**
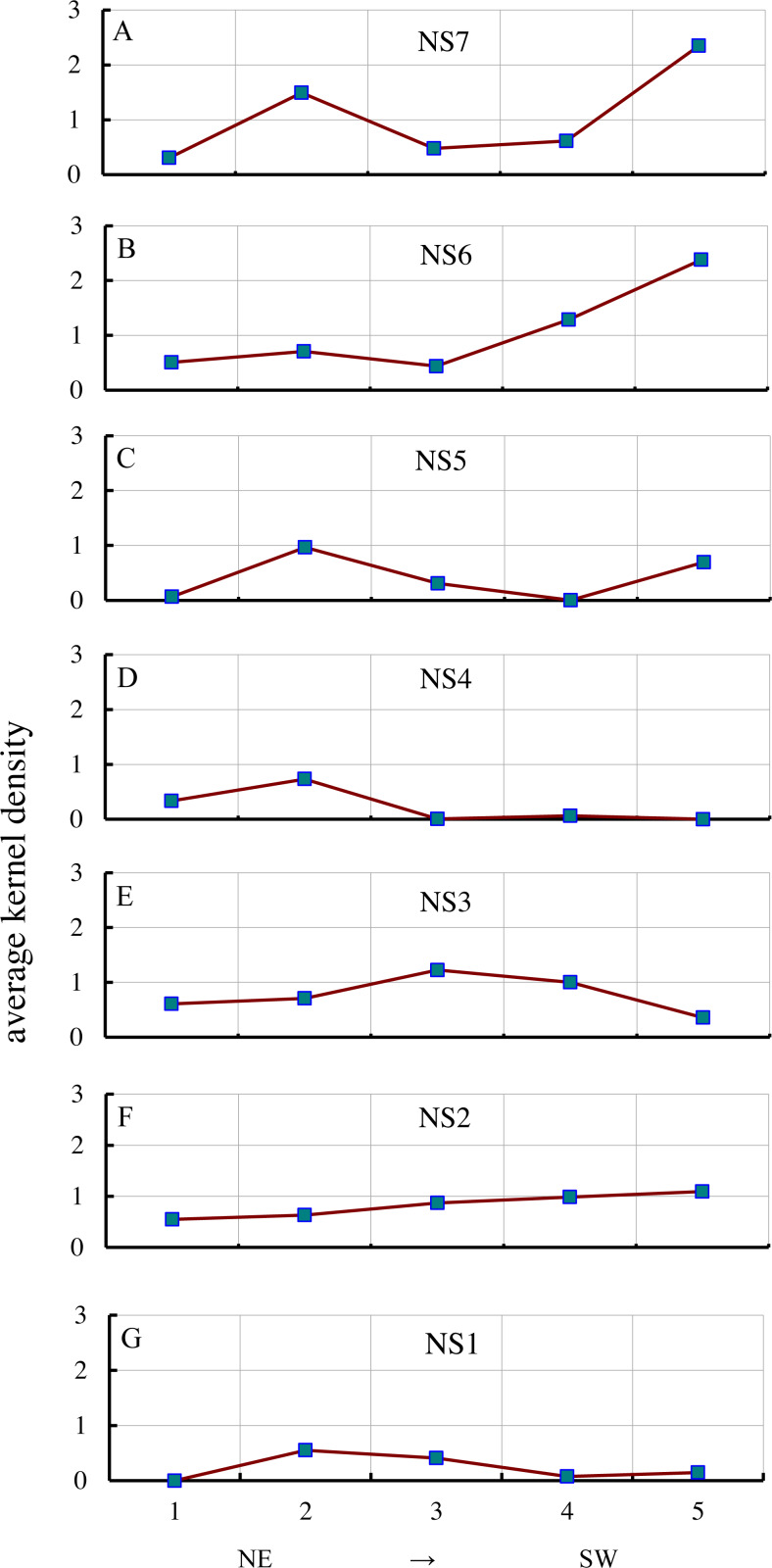
Variations in AKD from NE to SW. A, B, C, D, E, F & G represent NS7, NS6, NS5, NS4, NS3, NS2 and NS1, respectively, indicating the AKD change from the NE to SW of a near north-south belt transect.

The AKD of gullies changed from the northeast to the southwest (Fig. 6). The three series in the east (NS5, NS6, NS7) had low values in the middle and high values at the two sides. AKD in the northeast and southwest was relatively high, while in the center it was relatively low. In the eastern sample area series (NS7), from northeast to southwest, the first sample area (S57) had a low AKD of 0.30, which increased to 1.49 (S47), then fell to 0.48 (S37), and then increased from 0.62 (S27) to 2.35 (S17). The AKD of gullies in the southwest was significantly higher (1.6 times) than in the northeast. From east to west, the second series of the sample areas (NS6) had almost the same pattern of changes as the easternmost series (NS7), but the change in AKD of the three sample areas (S56, S46, and S36) in the northeast was relatively small, and the difference between their maximum value (0.71) and the maximum value (2.38) in the southwest was relatively large, i.e., 3.4 times larger. In the third sample area series (NS5), the changes in AKD followed the same pattern as those of the easternmost series (NS7), but the maximum value (0.70) in the southwest was lower than the maximum value in the northeast (0.97). In the fourth sample series (NS4), the AKD values of the three sample areas (S34, S24, and S14) in the southwest were lowest, but the AKD of the S24 sample area was slightly higher than that of the two adjacent sample areas. The maximum value of AKD (0.062) in the southwest was significantly (11.8 times) lower than in the northeast (0.73). There was a large difference between the pattern of changes in the three westernmost series (NS1, NS2, NS3) and the three easternmost series (NS5, NS6, NS7). In the fifth sample area series (NS3), the values in the middle were high, while the values at the two sides were low, i.e., AKD in the southwest and northeast was relatively low, while the values in the central area were relatively high. The maximum value of AKD in the middle was 1.23, and the minimum value in the southwest (0.36) was lower than the minimum value in the northeast (0.61). In the sixth sample area series (NS2), a gradual change was apparent, with a gradual increase from the minimum value in the northeast (0.55) to the maximum value in the southwest (1.10). In the seventh sample area series (NS1), AKD in the middle was low, but it was high at the two sides; however, AKD in the four sample areas was lower. The minimum value in the middle was 0.08, and the maximum value in the southwest (0.15) was lower than the maximum value in the northeast (0.55).

In general, the KD of gullies in northeastern China did not change monotonically in the horizontal direction. From the regional distribution of AKD, it was found that the AKD of the plains with a flat topography was lower, and gullies did not well develop. The areas with a higher AKD were distributed mainly in the transitional belt from mountains to plains or in the lower mountains and hills. The sample areas with the highest AKD were S16 (South Fushun City, 2.38) and S17 (east of Kuandian Manzu Autonomous County, 2.35), which were distributed in hilly areas or lower mountains and hills. The main landscape type of S47 (south of Linkou County, 1.49), which also had a high value in hilly areas. The areas with the lowest AKD values were S14 (west of Tongliao City, 0.001), S24 (northeast of Tongyu County, 0.06), and S34 (northwest of Daqing City, 0.005), which were located in Songliao Plain, Song-nen Plain, and Nenjiang Plain, respectively. There were also low values in S11 (southeast of Xin Barag Left Banner, 0.15) and S21 (west of Prairie Chenbarhu Banner, 0.08), which are located in the Hulun Buir Plateau. In general, the spatial clustering pattern of gullies in northeastern China follows a basic pattern of a low degree of clustering in the central area and a high degree of clustering on the three surrounding sides, which is similar to the terrain pattern in northeastern China.

### Vertical differentiation

#### Changes with mean elevation

A correlation analysis was performed on AKD and the mean elevation of the 34 sample areas. The Spearman correlation coefficient was 0.36 and the *P* value was 0.04; hence, the correlation was weak. The mean elevation was divided into seven levels: 0–200 m, 200–400 m, 400–600 m, 600–800 m, 800–1000 m, 1000–1200 m, and 1200–1400 m.

**Figure 7:**
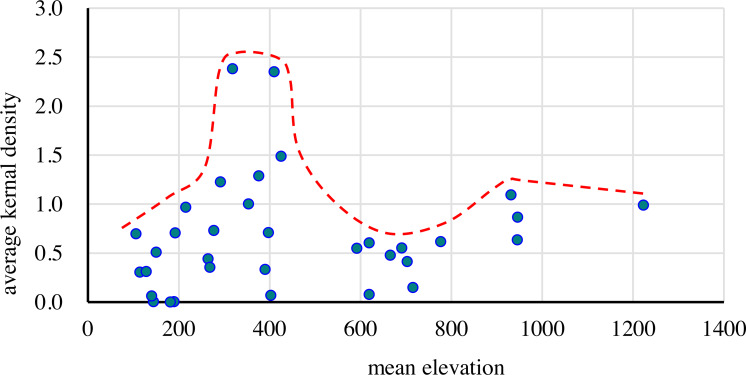
Distribution of average kernel density (AKD) with mean elevation.

From Fig. 7, it can be seen that the changes in AKD with elevation were complex. The maximum value of AKD in the group of mean height first increased rapidly, then decreased suddenly, and then gently increased followed by a gentle decline, i.e., it was low in the middle and high on both sides as the dotted red line represent. This pattern was particularly pronounced in low-altitude areas where the changes in gully density were more significant. There were two regions (second to the third levels and the fifth level) with a high AKD, but the maximum value of the second and third levels (2.38) was significantly higher (2.16 times) than that of the fifth level (1.10). In the first elevation level, AKD in the range of 100–200 m was in the range 0–0.71, the degree of clustering was low, and the gullies were sparsely distributed. In the second elevation level, AKD was between 0.33 and 2.38, and the value changed dramatically, indicating that there were both high-density areas and extremely sparse areas. In the third elevation level, AKD was between 0.01 and 2.35, with a distribution the same as that of the second elevation zone. In the fourth elevation level, AKD was between 0.08 and 0.62, which indicated that the spatial distribution of gullies was relatively uniform. In the fifth elevation level, AKD was between 0.63 and 1.10, indicating a relatively dense distribution of gullies. There were no samples in the sixth elevation level. In the seventh elevation level, AKD was 0.99. The sample areas with low AKD values (<0.5) were distributed mainly in an elevation range of 100–700 m, with the medium values (0.5–1.2) being spread over a wide elevation range and the high values (>1.2) concentrated in the range of 300–500 m.

#### Changes with height difference

A correlation analysis was conducted on the AKD values and height differences of the 34 sample areas. The Spearman correlation coefficient was 0.503 and the *P* value was 0.002, indicating a significant correlation between them. AKD also increased with an increase in the height difference. This trend could be fitted with a power function: *y* = 0.00009*x*^1.4272^. Based on 200 m intervals, the height difference could be divided into six levels: 0–200 m, 200–400 m, 400–600 m, 600–800 m, 800–1,000 m, and 1,000–1,200 m.

**Figure 8:**
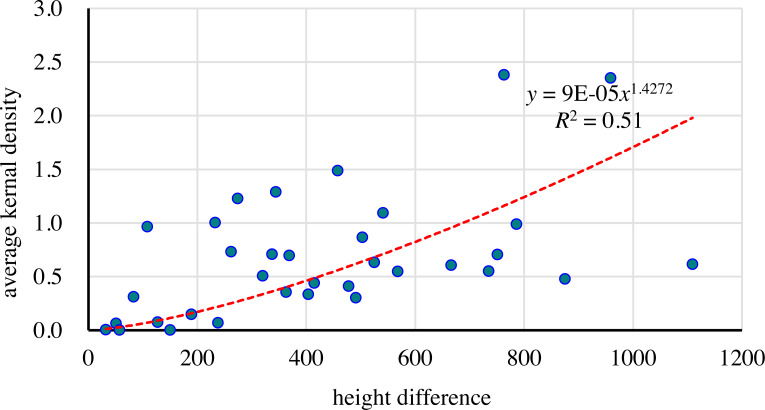
Relationship between height difference and AKD.

As the height difference increased, the maximum and minimum AKD in the interval that is every 200 m of the height difference increased significantly (Fig. 8). With the exception of S45, which had an AKD of 0.97, the AKD in the first level samples was between 0 and 0.31, which indicated that the gullies were not well developed in that area and their distribution was generally sparse. In the second level, AKD was between 0.07 and 1.29. In the third level, AKD was between 0.30 and 1.49. In the fourth level, AKD was concentrated mainly between 0.55 and 0.99, but with a high-value point of 2.38. In the fifth level, there were only two sample areas, with values of 0.48 and 2.35, respectively. In the sixth level, there was only one sample area, with a value of 0.62. The sample areas with a low AKD (<0.5) were distributed in areas with a height difference of less than 500 m. The sample areas with a medium AKD (0.5–1.2) were concentrated in the height difference range of 300–800 m, and the sample areas with a high AKD were distributed mainly in areas with a height difference of 200–600 m. With an increase in the height difference, the AKD spacing in each interval increased. This indicated that, with an increase in the height difference, the intensity of gully development displayed an increasing trend, and the degree of influence by various factors may increase.

Therefore, the AKD value of gullies was relatively low, and gullies were relatively underdeveloped in low- and high-altitude areas, while mid-altitude areas may be more prone to the development of gullies. Compared with elevation, the influence of the height difference on the development of gullies was more significant, and an increase in the height difference may be more conducive to their development. There is a complex mechanism that controls the relationship between height difference and the development of gullies. The height difference not only affects slope, vegetation distribution, and precipitation, but also has a large influence on hydrodynamic conditions.

## Discussion

### Azonality of gully distribution

Zonality refers to the regularity of the distribution of the elements of the natural environment along the surface of a strip of land and tapering in a certain direction. Climate, hydrology, vegetation, and soil are zonal factors, with climate being the dominant zonal factor (Sieben, 2019). Land–sea distribution, topography, and rocks are all azonal factors, with topography being the dominant azonal factor. In our chosen study area, the horizontal direction of AKD had a pattern of low values in the middle- and high-elevation zone at the three mountainous areas (north, east and west). AKD in the plains was extremely low, while in the surrounding mountainous and marginal areas (hills and platforms) it was higher. A similar pattern was observed in a study of the relationship between geomorphic type and gullies by Yan et al. (2005) in the Kebai County of northeastern China. That study found that the greatest concentration of gullies was to be found in the hilly area and the lowest density in the plain area, while in the platform area between the two areas, the density and extent of gullies was largest in the low-hill area (Yan et al., 2005). This indicates that the distribution of gullies in the northeastern region is restricted by the landform and azonal features exhibited in the horizontal direction. The spatial distribution of gullies in our study was also consistent with the results of Yang et al. (2017).

The vertical differentiation of AKD with elevation indicated a pattern with an initial increase followed by a decrease. The highest AKD values were generally distributed between 200 and 410 m, which was similar to the distribution of the maximum gully density with elevation at the small regional scale. For example, the gully density in the black soil area of the Wuyuer River and the Namoer River Basin is between 250 and 280 m. In a study of the black soil area in the eastern part of Kebai, the gully density peaked between 210 and 280 m (Yan, Zhang & Yue, 2007). In a study of the Wuyuer River Basin, the elevation zone with the highest rate of soil erosion was located between 200 and 250 m (E, 2015). Gully erosion in different altitudes was affected by human activities to different degrees. The intensity of human activities is greater in lower altitudes, especially in cultivated areas, because cultivation tends to fill ephemeral gullies, thus eliminating them. Although the intensity of human activities is low and the vegetation coverage is high in the higher altitude areas, gully erosion is not easy to occur. Therefore, gully erosion is easy to occur in the slope areas with moderate altitude (Yan, Zhang & Yue, 2007). Although the scales of the different study areas varied considerably, the peak gully density varied with elevation at each different scale, and the general trends were consistent.

### Diverse factors influencing gully distribution

In addition to the geomorphic types, the distribution of gullies in the study area was also influenced by precipitation, vegetation, soil, and other factors. The annual rainfall in northeastern China gradually decreases from southeast to northwest, which is consistent with the trend of decreasing soil erosion from southeast to northwest (Jiao, 2010). Although the southern part of the Changbai Mountains and the northeastern part of the Greater Khingan Range have a similar landform, gullies are more developed in the southeast than in the northwest due to the difference in precipitation. Vegetation coverage in northeastern China is extensive, but it gradually decreases from east to west, with the characteristics of high coverage in the east and low coverage in the west (Hu, Liu & Mao, 2017). This is inconsistent with the general trend of gully density. Although the vegetation coverage in the western part of the study area was low, the precipitation was also low, and water erosion was therefore relatively weak, while wind erosion was stronger. Gullies in this area were therefore rare. In addition to vegetation coverage, human activities can lead to changes in land-use types. The expansion of cultivated land and the reduction in grassland and forestland can exacerbate soil erosion. There are diverse soil types in northeastern China, including black soil, chernozem, meadow soil, and planosol. The cultivated areas are dominated by black soil (Fan et al., 2018). Because soils in different regions are affected by topography, climate, vegetation, and human activities, the soils vary in physical and chemical properties, with differences in soil organic matter content and soil anti-erodibility properties facilitating soil erosion. The greater topographic relief and higher annual rainfall in the Liaodong Hills resulted in a dense gully distribution (S16 and S17). The S47 sample area is located in the eastern hilly area of the Mudanjiang River Basin in the Changbai Mountains, where the geological structure in the semi-mountainous area consists of relatively loose rocks; short-term blizzards and heavy rains are common there, leading to serious soil erosion (Zhang et al., 2017). The locations of S22, S23, and S33 are in the transition zone from the Greater Khingan Range to the Song-nen Plain, with a large variation in topographic relief and a strong freeze–thaw effect, resulting in relatively serious gully erosion. Human activities in the sample areas have led to changes in the land-use types and the destruction of vegetation (Zhang, Zhang & Liu, 2006), which is another reason why the AKD is higher in these areas than in other areas with similar geomorphic types. Therefore, in different regions, the dominant factors leading to the development of gullies may be different. There is a need to further understand how topography, climate, vegetation, and soil all control the development of gullies and how much each factor contributes to the overall pattern of gully distribution. In particular, a quantitative expression of these factors should be considered in future studies.

### Effectiveness of the KDE

As the most common method of hot spot analysis, the KDE provides a good quantitative tool for determining what controls the distribution of gullies, which can reveal the trends in their continuous spatial distribution. The KDE can be smoothly calculated and effectively eliminate the impact of boundary separation on density estimation , overcoming the homogeneity of a gully density distribution calculated by subjective boundaries (Golosov et al., 2018; Zhao et al., 2016) and abrupt changes in sampling intervals. Unlike the point density method, the methods used in this study considered the length of gullies, but did not consider the width or area of gullies. Gullies in cultivated areas often have obvious edge lines (often U-shaped gullies), which can be extracted easily from Google Earth images. However, when there is no obvious gully edge line (often V-shaped gullies in mountainous areas), it cannot be extracted. If we can get the gullies with area (even 3D gully), we can get better results of spatial pattern of gullies. It can be achieved in a small region, but very difficult in a large region. If the depth or volume of gullies were to be considered, the evaluation of the three-dimensional properties of a gully could provide more precise information on gully volume. However, obtaining accurate three-dimensional gully parameters is technically feasible at the regional scale, but is difficult to achieve in terms of the time involved and the economic cost. This would require technological advances, especially the development of stereoscopic technology for high-resolution remote sensing images, which would provide a more detailed and reliable data source for gully research. In addition, the establishment of evaluation techniques for gully activity and a better understanding of the spatial clustering of gullies with different degrees of activity would provide a scientific basis for gully control.

## Conclusions

In this study, 34 sample areas were selected in northeastern China, and a kernel density evaluation (KDE) was conducted in order to explore the spatial distribution of gullies in these areas. According to the minimum, maximum, and average values of kernel density (KD), the gullies in the 34 sample areas displayed obvious differences and had an inhomogeneous distribution. The average kernel density (AKD) was used as a key index to characterize the spatial pattern of gully distribution. In terms of spatial azonality, the AKD of the 34 sample areas indicated a clustering pattern, and the spatial distribution of AKD had the characteristics of a concentrated distribution of high- and low-value areas. From the perspective of horizontal differentiation, AKD was higher in the transition zone from west, north, and east to the plains or in the low mountains and hills, while it was lower in the plains. The horizontal distribution of gullies showed a basic pattern of low clustering in the middle and high clustering in the three mountainous areas (north, east and west), which was similar to the geomorphic pattern in Northeastern China. From the perspective of vertical differentiation, the changes in AKD were more complex with increasing elevation, with values being generally low in the center and high at both sides, indicating that gullies were relatively underdeveloped in low- and high-altitude areas, while mid-altitude areas may be prone to the development of gullies. The maximum and minimum values of AKD in the interval that is every 200 m of the height difference increased with increasing height difference, which means that the latter may lead to the development of gullies. The variations in AKD reflect the changes in the spatial clustering characteristics of gullies, indicating that spatial clustering is related to the geomorphic pattern, elevation, and height difference, and reflects the interactions among these factors that control gully formation. This study not only revealed the spatial clustering characteristics of gullies in northeastern China but also provided a scientific reference for future studies of water and soil conservation.

##  Supplemental Information

10.7717/peerj.9907/supp-1 Supplemental Information 1 Raw date for Figs. 5– 8

## Additional Information and Declarations

### Data Availability

The following information was supplied regarding data availability: The raw data are available in the Supplemental Files.

## References

[ref-1] Arabameri A, Rezaei K, Pourghasemi HR, Lee S, Yamani M (2018). GIS-based gully erosion susceptibility mapping: a comparison among three data-driven models and AHP knowledge-based technique. Environmental Earth Sciences.

[ref-2] Bogale A, Aynalem D, Adem A, Mekuria W, Tilahun S (2020). Spatial and temporal variability of soil loss in gully erosion in upper Blue Nile basin, Ethiopia. Applied Water Science.

[ref-3] Chai Z, Fan J, Liu S (2001). Analysis on development characteristics and process of gully in Yuanmon Basin on lower reaches of Jinsha River. Scientia Geographica Sinica.

[ref-4] Chen D, Zhang S, Wang R, Pu L, Chang L, Yang J (2016). Study on gully erosion distribution in Northeast black soil areas based on Pleiades. Journal of Northeast Normal University (Natural Science Edition).

[ref-5] Deng Q, Liu H, Cheng W, Yang H, Liu G, Luo J, Qin F, Yang D, Zhang B (2020). Experimental investigations of the evolution of step-pools in rills with heterogeneous soils in Yuanmou Dry-Hot Valley, SW China. Catena.

[ref-6] Deng Q, Qin F, Zhang B, Wang H, Luo M, Shu C, Liu H, Liu G (2015). Characterizing the morphology of gully cross-sections based on PCA: a case of Yuanmou Dry-Hot Valley. Geomorphology.

[ref-7] Dong Y, Nie Y, Xiong D (2018). Investigation of gully density and classification of Hengduan Mountainous area based on Google Earth images. Bulletin of Soil and Water Conservation.

[ref-8] E L (2015). Study on spatial temporal patterns and contributory factors of soil erosion in Wuyur River Basin. Master’s thesis.

[ref-9] Fan H, Zhen F, Wang Y (2015). Influence of rainfall characteristics on distribution of gullies in Heilongjiang province. Technology of Soil and Water Conservation.

[ref-10] Fan Z, Peng C, Jin R, Wu H (2018). Main soil types in Northeast China and fertility index correlation with meteorological factors. Journal of Maize Sciences.

[ref-11] Frankl A, Zwertvaegher A, Poesen J, Nyssen J (2013). Transferring Google Earth observations to GIS-software: example from gully erosion study. International Journal of Digital Earth.

[ref-12] Gao X, Wu P, Zhao X, Wang J, Shi Y, Zhang B, Tian L, Li H (2013). Estimation of spatial soil moisture averages in a large gully of the Loess Plateau of China through statistical and modeling solutions. Journal of Hydrology.

[ref-13] Golosov V, Yermolaev O, Rysin I, Vanmaercke M, Medvedeva R, Zaytseva M (2018). Mapping and spatial–temporal assessment of gully density in the Middle Volga region, Russia. Earth Surface Processes and Landforms.

[ref-14] Gurbanov EA, Ganieva SA (2017). Intensity of gully erosion in arid zone of Azerbaijan republic (by the example of the region of the Mingechaur water reservoir). Arid Ecosystems.

[ref-15] Guyassa E, Frankl A, Zenebe A, Poesen J, Nyssen J (2017). Effects of check dams on runoff characteristics along gully reaches, the case of Northern Ethiopia. Journal of Hydrology.

[ref-16] Guyassa E, Frankl A, Zenebe A, Poesen J, Nyssen J (2018). Gully and soil and water conservation structure densities in semi-arid northern Ethiopia over the last 80 years. Earth Surface Processes and Landforms.

[ref-17] Hu S, Liu J, Mao X (2017). Extraction and change of vegetation coverage of 2007-2010 in Northeast China. Journal of Northeast Forestry University.

[ref-18] Huang M, Fan H (2017). Development characteristics and topographic differentiation features of erosion gully in Liaoning Province of China. Journal of Soil and Water Conservation.

[ref-19] Jiao J (2010). Study on spatial variation of soil erosion in Northeastern China. Reserch of Soil and Water Conservation.

[ref-20] Karydas C, Panagos P (2020). Towards an assessment of the ephemeral gully erosion potential in Greece using Google Earth. Water.

[ref-21] Kumari M, Sarma K, Sharma R (2019). Using Moran’s I and GIS to study the spatial pattern of land surface temperature in relation to land use/cover around a thermal power plant in Singrauli district, Madhya Pradesh, India. Remote Sensing Applications: Society and Environment.

[ref-22] Li R, Li Y, Wen W, Zhou Y (2017). Comparative study on spatial difference of elevation and slope in soil erosion evolution in typical watershed. Journal of Soil and Water Conservation.

[ref-23] Li X, Wang Z, Zhang S, Yan Y (2007). Dynamics and spatial distribution of gully in the typical upland region of Northeast China. Scientia Geographica Sinica.

[ref-24] Li F, Zhang S, Li T (2012). The spatial distribution relations between erosion gully and terrain factors in the south oftypical black soil zone in Northeast China. Soil and Crop.

[ref-25] Li X, Zhao C, Zhou X (2019). Vegetation pattern of Northeast China during the special periods since the Last Glacial Maximum. Scientia Sinica(Terrae).

[ref-26] Litvin LF, Zorina YF, Sidorchuk AY, Chernov AV, Golosov VN (2003). Erosion and sedimentation on the Russian Plain, part 1: contemporary processes. Hydrological Processes.

[ref-27] Luffman I, Nandi A (2020). Seasonal Precipitation Variability and Gully Erosion in Southeastern USA. Water.

[ref-28] Okwu-Delunzu VU, Enete IC, Abubakar AS, Lamidi S, Neale CMU, Maltese A (2013). Monitoring gully erosion at Nyaba River of Enugu State Southeastern Nigeria, using remote sensing. Remote sensing for agriculture, ecosystems, and hydrology Xv.

[ref-29] Parzen E (1962). On estimation of a probability density function and mode. Annals of Mathematical Statistics.

[ref-30] Poesen J, Nachtergaele J, Verstraeten G, Valentin C (2003). Gully erosion and environmental change: importance and research needs. Catena.

[ref-31] Rahmati O, Tahmasebipour N, Haghizadeh A, Pourghasemi HR, Feizizadeh B (2017). Evaluating the influence of geo-environmental factors on gully erosion in a semi-arid region of Iran: an integrated framework. Science of the Total Environment.

[ref-32] Ran H, Deng Q, Zhang B, Liu H, Wang L, Luo M, Qin F (2018). Morphology and influencing factors of rills in the steep slope in Yuanmou Dry-Hot Valley (SW China). Catena.

[ref-33] Rosenblatt M (1956). Remarks on some nonparametric estimates of a density function. Annals of Mathematical Statistics.

[ref-34] Ruppert D, Cline DBH (1994). Bias reduction in kernel density estimation by smoothed empirical transformations. Annals of Statistics.

[ref-35] Sieben EJJ (2019). Zonal and azonal vegetation revisited: how is wetland vegetation distributed across different zonobiomes. Austral Ecology.

[ref-36] Tang G, Li F, Yang X, Xiong L (2015). Exploration and practice of digital terrain analysis in the Loess Plateau.

[ref-37] Tian J, Tang G, Zhou Y, Song X (2013). Spatial variation of gully density in the loess plateau. Scientia Geographica Sinica.

[ref-38] Valentin C, Poesen J, Li Y (2005). Gully erosion: impacts, factors and control. Catena.

[ref-39] Wang D, Fan H (2019). Distribution characteristics of gullies with slope gradient in Northeast China. Environmental Monitoring and Assessment.

[ref-40] Wang D, Fan H, Fan X (2017). Distributions of recent gullies on hillslopes with different slopes and aspects in the Black Soil Region of Northeast China. Environmental Monitoring and Assessment.

[ref-41] Wang W, Zhang S, Li Y, Bu K, Yan Y (2008). Application of high-resolution images on soil loss quantitative estimation—a case of quickBird. System Sciences and Comprehensive Studies in Agriculture.

[ref-42] Wang R, Zhang S, Pu L, Yang J, Yang C, Chen J, Guan C, Wang Q, Chen D, Fu B, Sang X (2016). Gully erosion mapping and monitoring at multiple scales based on multi-source remote sensing data of the sancha river catchment, Northeast China. Isprs International Journal of Geo-Information.

[ref-43] Wu Y, Zheng Q, Zhang Y, Liu B, Cheng H, Wang Y (2008). Development of gullies and sediment production in the black soil region of northeastern China. Geomorphology.

[ref-44] Xu W (1986). The relation between the zonal distribution of types of vegetation and the climate in Northeast China. Acta Phytoecologica Et Geobotanica Sinica.

[ref-45] Xu X, Sui Y, Zhang Y, Ou Y, Luo L, Li Y (2014). Development of gully erosion and its influencing factors in hilly regions of Northeast China. Acta Pedologica Sinica.

[ref-46] Xu Z, Qin FC, Zhang B, Deng QC, Liu H, Jin J, Shi LT (2018). The morphological characteristics of gully systems and watersheds in Dry-Hot Valley, SW China. Acta Geochimica.

[ref-47] Yang J, Zhang S, Chang L, Li F, Li T, Gao Y (2017). Gully erosion regionalization of black soil area in northeastern China. Chinese Geographical Science.

[ref-48] Yan Y, Zhang S, Li X, Yue S (2005). Temporal and spatial variation of erosion gullies in kebai black soil region of Heilongjiang during the past 50 years. Acta Geographica Sinica.

[ref-49] Yan Y, Zhang S, Yue S (2006). Application of corona and spot imagery on erosion gully research in typical black soil regions of Northeast China. Resources Science.

[ref-50] Yan Y, Zhang S, Yue S (2007). Classification of erosion gullies by remote sensing and spatial pattern analysis in black soil region of Eastern Kebai. Scientia Geographica Sinica.

[ref-51] Yi Q, Zhang Y, Zhang H, Zhang P, Ma S, Yao G (2016). Study on the spatial distribution characteristics of the eroded gully in gullied rolling loess area of West Henan province. Yellow River.

[ref-52] Zhan X, Lin A, Sun C, Qiao W (2016). Centrality of public transportation network and its coupling with bank branches distribution in Wuha. Progress in Geography.

[ref-53] Zhang S, Li F, Li T, Yang J, Bu K, Chang L, Wang W, Yan Y (2015). Remote sensing monitoring of gullies on a regional scale: A case study of Kebai region in Heilongjiang Province, China. Chinese Geographical Science.

[ref-54] Zhang Y, Zhang S, Liu Z (2006). Analyzing land use/cover changes in the upper reaches of taoerhe river catchments. Resources Science.

[ref-55] Zhang L, Zhang X, Ni Y, Zhou H (2017). Study on the division of the geographic sensitive area in Mudanjiang River Basin. Environmental Protection and Circular Economy.

[ref-56] Zhao J, Vanmaercke M, Chen L, Govers G (2016). Vegetation cover and topography rather than human disturbance control gully density and sediment production on the Chinese Loess Plateau. Geomorphology.

